# MRI with hyperpolarised [1-^13^C]pyruvate detects advanced pancreatic preneoplasia prior to invasive disease in a mouse model

**DOI:** 10.1136/gutjnl-2015-310114

**Published:** 2015-09-07

**Authors:** Eva M Serrao, Mikko I Kettunen, Tiago B Rodrigues, Piotr Dzien, Alan J Wright, Aarthi Gopinathan, Ferdia A Gallagher, David Y Lewis, Kristopher K Frese, Jaime Almeida, William J Howat, David A Tuveson, Kevin M Brindle

**Affiliations:** 1 Cancer Research UK Cambridge Institute, University of Cambridge, Cambridge, UK; 2 Department of Biochemistry, University of Cambridge, Cambridge, UK; 3 A.I.Virtanen Institute for Molecular Sciences, University of Eastern Finland, Kuopio, Finland; 4 Department of Radiology, University of Cambridge, Cambridge, UK; 5 Princess Margaret Cancer Centre, Toronto, Ontario, Canada; 6 Cold Spring Harbor Laboratory, New York, New York, USA

**Keywords:** CANCER, MAGNETIC RESONANCE IMAGING, PANCREAS

## Abstract

**Objectives:**

Pancreatic cancer (PCa) is treatable by surgery when detected at an early stage. Non-invasive imaging methods able to detect both established tumours and their precursor lesions are needed to select patients for surgery. We investigated here whether pancreatic preneoplasia could be detected prior to the development of invasive cancers in genetically engineered mouse models of PCa using metabolic imaging.

**Design:**

The concentrations of alanine and lactate and the activities of lactate dehydrogenase (LDH) and alanine aminotransferase (ALT) were measured in extracts prepared from the pancreas of animals at different stages of disease progression; from pancreatitis, through tissue with predominantly low-grade and then high-grade pancreatic intraepithelial neoplasia and then tumour. ^13^C magnetic resonance spectroscopic imaging (^13^C-MRSI) was used to measure non-invasively changes in ^13^C labelling of alanine and lactate with disease progression, following injection of hyperpolarised [1-^13^C]pyruvate.

**Results:**

Progressive decreases in the alanine/lactate concentration ratio and ALT/LDH activity ratio with disease progression were accompanied by a corresponding decrease in the [1-^13^C]alanine/[1-^13^C]lactate signal ratio observed in ^13^C-MRSI images of the pancreas.

**Conclusions:**

Metabolic imaging with hyperpolarised [1-^13^C]pyruvate enables detection and monitoring of the progression of PCa precursor lesions. Translation of this MRI technique to the clinic has the potential to improve the management of patients at high risk of developing PCa.

**Significance of this study**

**What is already known on this subject?**

- Pancreatic cancer is one of the leading causes of cancer-related deaths. If detected at an early stage, it is potentially curable.
- New diagnostic techniques are urgently required for the detection of early-stage pancreatic cancer.
- Application of dynamic nuclear polarisation has increased the sensitivity of magnetic resonance spectroscopy by more than 10 000-fold, allowing real-time imaging of tissue metabolism in vivo.
- Hyperpolarised [1-^13^C]pyruvate has been used recently in humans for the first time to detect tumours in the prostate.

**What are the new findings?**

- Progression of pancreatic cancer from tissue containing predominantly low-grade precursor lesions to tissue containing tumour showed a decreasing alanine/lactate concentration ratio and alanine aminotransferase (ALT)/lactate dehydrogenase (LDH) activity ratio in tissue extracts.
- ^13^C-magnetic resonance spectroscopic images of the pancreas following injection of hyperpolarised [1-^13^C]pyruvate showed a decrease in the [1-^13^C]alanine/[1-^13^C]lactate signal ratio with disease progression, which was explained by the changes in alanine and lactate concentrations and LDH and ALT activities.
- Metabolic imaging with hyperpolarised [1-^13^C]pyruvate can be used to detect and monitor the progression of pancreatic cancer precursor lesions in a mouse model of the human disease.

**How might it impact on clinical practice in the foreseeable future?**

- ^13^C-magnetic resonance spectroscopic imaging with hyperpolarised [1-^13^C]pyruvate may allow a non-invasive, radiation-free method for early detection of disease progression in individuals at high risk of developing pancreatic cancer, allowing curative intervention to be offered at an earlier stage and consequently improving patient survival and prognosis.

## Introduction

Pancreatic cancer (PCa) is the fourth leading cause of cancer-related deaths, with approximately equal rates of annual incidence and mortality.^1^ The 5-year survival rate has remained at 5–6% for the last four decades.^2^ ^3^ At the time of diagnosis, >80% of patients are ineligible for curative surgical treatment, and of those amenable to surgery, the majority will relapse.^4^ Late clinical presentation, inaccurate early diagnosis using current biomarkers and imaging methods, limited treatment options and drug resistance continue to make PCa difficult to treat.^5^ Since PCa is potentially curable by surgery, the best option to improve survival rates would be to increase the number of candidates for surgery through early detection of disease progression.

Pancreatic ductal adenocarcinoma (PDA) evolves through a spectrum of intraepithelial neoplasia (PanIN) precursor lesions as a result of accumulating mutations. PanIN1 and PanIN2 can occur in normal individuals and in chronic pancreatitis without ever evolving into PDA, whereas PanIN3, termed carcinoma in situ, shares many of the genetic alterations of PDA and has a greater potential to evolve into invasive PDA.^6–10^ Between 10% and 36% of those diagnosed with PCa have a genetic predisposition, for example, individuals with familial pancreatic cancer (FPC), and selective screening of this population is recommended.^11–13^ However, identification of curable precursor lesions of PDA in these individuals is still unsatisfactory. Non-invasive proton magnetic resonance spectroscopy (^1^H-MRS) measurements of tissue metabolites have been limited by low sensitivity and masking of the lactate proton signal by intense overlapping lipid resonances, with the few studies that have been performed focusing on the differentiation of PCa from pancreatitis and normal tissue.^14^ ^15^ The only clinically available serum biomarker, CA19.9, is of limited use, cross-sectional imaging techniques are unreliable and invasive endoscopic procedures are operator dependent and can give unclear and false-positive findings.^11^ ^16^ ^17^ Thus, there is an urgent need for the development of new and better diagnostic methods.^11^

^13^C magnetic resonance spectroscopic imaging (^13^C-MRSI) using ^13^C-labelled substrates, in which the ^13^C nuclear spins have been hyperpolarised using dynamic nuclear polarisation, has revolutionised metabolic imaging with MR by increasing the sensitivity of detection by >10 000-fold.^18^ This has allowed imaging of hyperpolarised ^13^C labelled substrates and the metabolites formed from them in vivo.^19^ Hyperpolarised [1-^13^C]pyruvate has been the most widely used substrate, having shown considerable promise in preclinical studies for tumour grading and assessment of treatment response.^19^ ^20^

We show here, in genetically engineered mouse models that recapitulate many of the clinical, histopathological, genetic and metabolic aspects of the human disease, that hyperpolarised [1-^13^C]pyruvate has the potential to detect and follow the progression of pancreatic precursor lesions towards invasive PCa.^21–24^

## Materials and methods

### Animal preparation

Experiments complied with licences issued under the Animals (Scientific Procedures) Act of 1986. Protocols were approved by the Cancer Research UK, Cambridge Institute Animal Welfare and Ethical Review Body. LSL-Kras^G12D/+^- p48^Cre/+^ (KC) mice (2, 4 and 9 months old) with mPanIN lesions, LSL-Kras^G12/D+;^ LSL-Tpr53^R172H/+;^Pdx-1-Cre (KPC) mice (3–6 months old) with spontaneous PCa, LSL-Kras^G12D/+^ and p48^Cre/+^ control mice (age matched to KC mice), Pdx-1-Cre (PC) control mice (age matched to KPC) and C57BL/6 control wild-type (wt) mice, with no pancreatic lesions (6–8 weeks old) were used.^23^ Tumours were studied at <5 mm diameter. Acute pancreatitis was induced in wt mice by six hourly intraperitoneal injections with 50 μg/kg of caerulein (Sigma-Aldrich, Dorset, UK).^25^

### Hyperpolarisation of [1-^13^C]pyruvate

[1-^13^C]Pyruvic acid samples (44 mg, 14 mol/L; 99% ^13^C) containing 15 mmol/L of trityl radical, tris (8-carboxy-2,2,6,6-tetra-(hydroxyethyl)-benzo-[1,2–4,5]-bis-(1,3)-dithiole-4-yl)-methyl sodium salt (OX063; GE Healthcare, Amersham, UK) and 1.5 mmol/L of an aqueous solution of a gadolinium chelate (Dotarem, Guerbet, Roissy, France) were polarised in a Hypersense polariser (Oxford Instruments, Abingdon, UK). The frozen sample was dissolved at 180°C in 6 mL buffer containing 40 mM HEPES, 94 mM NaOH, 30 mM NaCl and 50 mg/L EDTA. Polarisation levels ranged from 16% to 25%, measured using a polarimeter (Oxford Instruments, UK).

### Magnetic resonance spectroscopy and imaging

Mice were anaesthetised by inhalation of 1–2% isoflurane (Isoflo, Abbotts Laboratories, Maidenhead, UK) in air/O_2_ (75/25% vol/vol, 2 L/min). Body temperature was maintained using warm air. Breathing rate (∼80 bpm) and body temperature (37°C) were monitored (Biotrig, Small Animal Instruments, Stony Brook, New York, USA). Experiments were performed in a 7.0-T horizontal bore magnet (Agilent, Palo Alto, California, USA) using an actively decoupled dual-tuned ^13^C/^1^H volume transmit coil (Rapid Biomedical, Rimpar, Germany) and a 20 mm diameter ^13^C receiver coil (Rapid Biomedical). The pancreas was localised using respiratory-gated coronal and axial T_2_-weighted fast spin-echo images (repetition time (TR) 2 s; echo time (TE) 12 ms; field of view (FOV) 80 mm×40 mm; data matrix 512×256; slice thickness 1.25 mm; 12 slices). Hyperpolarised [1-^13^C]pyruvate (0.3 mL, 82 mM) was injected intravenously, via a tail vein catheter, over a period of 3 s, and the animal placed inside the magnet. Axial ^13^C chemical-shift images (CSI) (TR 30 ms; TE 1.5 ms; FOV 40×40 mm; data matrix 32×32 with centre-out encoding order; spectral width 6 kHz; total acquisition time 30 s, flip angle 5°) were collected from the 4–8-mm-thick slices selected from the ^1^H images. Spectroscopic image acquisition commenced 20±2 s after the start of injection, with a total time between dissolution and data acquisition of ∼30 s. CSI analysis was performed in MATLAB (The Mathworks, Massachusetts, USA), by an independent blinded observer. The data were multiplied by a cosine function and zero-filled to 128 points in both spatial directions, line-broadened to 20 Hz and zero-filled to 1024 points in the spectral dimension before Fourier transformation, phase and baseline correction and peak integration. A total of 139 spectroscopic imaging examinations were performed in 93 mice. From these, 10 scans were excluded due to poor visualisation of the pancreas (n=3), poor signal-to-noise ratio in the ^13^C spectra (n=5) and death of the mouse following injection (n=2).

### Measurements of enzyme activities and metabolite concentrations

Mice were sacrificed by cervical dislocation and pancreatic tissue rapidly excised and freeze-clamped using liquid nitrogen-cooled tongs. Tissues were homogenised in radioimmunoprecipitation assay buffer (50 mM HEPES, 1 mM EDTA, 0.7% sodium deoxycholate, 1% Nonidet P-40, 0.5 M lithium chloride, pH 7.6), using a Precellys 24 homogeniser (Stretton Scientific, Stretton, UK). Homogenates were centrifuged and lactate dehydrogenase (LDH) and alanine aminotransferase (ALT) (Abcam, Ref. ab105134, Cambridge, UK) activities in the supernatant were assayed spectrophotometrically.^26^ Lactate and alanine concentrations were determined using ^1^H NMR spectroscopy. Tissues were extracted using a methanol:chloroform:water protocol, and high-resolution ^1^H and ^1^H-decoupled ^13^C NMR spectra were obtained at 14.1 T (25°C, pH 7.2) using a Bruker 600 MHz NMR spectrometer (Bruker, Ettlingen, Germany).^27^ The acquisition conditions were ^1^H, 90° pulses; 7.3 kHz spectral width; 4.5 s acquisition time; 32k data points; 64 transients; and 12.5 s recycling time; ^13^C, 30° pulses; 36.0 kHz spectral width; 0.9 s acquisition time; 32k data points; 2048 transients; and 14 s recycling time. Proton chemical shifts were referenced to 5 mM 3-(trimethylsilyl)-2,2′,3,3′-tetradeuteropropionic acid (TSP; 0.0 ppm), which was added to the samples. Peak integrals were analysed using ACD/SpecManager (ACD/Labs, Bracknell, UK). Data were zero-filled twice and multiplied by an exponential function prior to Fourier transformation. All ^1^H NMR resonance areas were normalised relative to the TSP resonance.

### ^1^H NMR measurements of ^13^C label exchange in pancreatic tissue extracts

Freeze-clamped pancreatic tissue was homogenised (1:2; g/mL) using a Precellys 24 homogeniser (Stretton Scientific, Stretton, UK) in a buffer designed to simulate the intracellular conditions. This contained 40 mM HEPES (pH 7.1) 10 mM nicotinamide, 2 mM dithiothreitol, 0.2 mM glutamate, 0.1 mM pyridoxal phosphate, 0.4 mM NAD^+^ and 0.2 M KCl at 37°C. Unlabelled alanine and lactate were added at concentrations equivalent to those measured in the respective tissues (table 1). ^13^C label incorporation from [3-^13^C]pyruvate into alanine and lactate was measured using ^1^H NMR from the splitting of their respective methyl proton resonances due to ^1^H-^13^C coupling. Measurements were made using a one-dimensional ^1^H-NOESY sequence with continuous-wave solvent saturation of 3.7 s in a total TR of 5.0 s with a mixing time of 0.15 s, 12.51 ppm spectral width and 8192 complex points. The sample temperature was maintained at 37°C. Spectra were analysed in MATLAB (The Mathworks) and used to calculate the concentrations of unlabelled and 3-^13^C labelled pyruvate, alanine and lactate at each time point. Rates of isotope exchange between pyruvate and alanine and lactate were calculated by fitting a linear function to the initial five points of the 3-^13^C alanine or lactate concentration curves. These rate constants, corrected for the effect of dilution of the tissue extract (wet weight (g) of homogenised tissue) in the NMR tube sample volume (mL), were used to calculate the extent of isotope exchange that would have been observed in vivo at 20 s after injection of hyperpolarised [1-^13^C]pyruvate.

**Table 1:** Lactate and alanine concentrations and lactate dehydrogenase and alanine aminotransferase activities in tissue extracts

Tissue	Alanine concentration (μmol/g of wet tissue)	Lactate concentration (μmol/g of wet tissue)	Lactate dehydrogenase activity (mU/mg of protein) (n=3)	Alanine aminotransferase activity (mU/mg of protein) (n=3)
Control pancreas (n=19)	1.82±0.32	1.55±0.24	204.35±53.1	6.10±0.35
Induced pancreatitis (n=12)	1.19±0.2	1.36±0.20	300.78±93.0	9.09±1.25
4-month-old pancreas (n=20)	0.98±0.11	1.80±0.20	616.44±100.05†(*)	5.09±1.32
9-month-old pancreas (n=16)	1.08±0.30	4.24±1.21	763.46±106.40†(**) ‡(*)	3.94±0.23
Tumour (sarcomatoid) (n=13)	4.58±1.00§(*) ¶(**)	17.80±4.1 §(***) †(****) ‡(****)	1008.9±49.25§(*) †(****) ‡(***)	0.28±0.01
Tumour (PDA) (n=12)	1.70±0.37	8.14±1.67 §(**) †(***) ‡(**)	1142.44±19.55 §(**) ¶(*) †(****) ‡(****)	0.17±0.04‡(*)

Pancreatic tissues from controls (LSL-Kras^G12D/+^ (n=4); p48^Cre^ (n=4); Pdx-1-Cre (PC) (n=6) and C57BL/6 wild-type (n=5)), 4-month-old and 9-month-old KC mice bearing mPanIN lesions and KPC mice with tumours. Mean±SEM.

*p<0.05, **p<0.01, ***p<0.001, ****p<0.0001.

†Significantly different compared with control pancreas.

‡Significantly different compared with induced pancreatitis.

§Significantly different compared with 4-month-old pancreas

¶Significantly different from 9-month-old pancreas.

KC, LSL-Kras^G12D/+^- p48^Cre/+^; KPC, LSL-Kras^G12/D+;^LSL-Tpr53^R172H/+;^Pdx-1-Cre; n, number of animals; PanIN, pancreatic intraepithelial neoplasia; PDA, pancreatic ductal adenocarcinoma.

### PET/CT

Clinical images were acquired 89 min after intravenous injection of 356 MBq of ^18^F-labelled fluorodeoxyglucose (^18^F-FDG). Mice were fasted overnight prior to intravenous administration of 5 MBq of ^18^F-FDG (IBA Molecular, Guildford, UK). Data were acquired between 60 and 90 min in list-mode format on a NanoPET/CT scanner (Mediso, Hungary). A CT image was acquired for anatomic registration. PET images were reconstructed using a two-dimensional ordered-subset expectation maximisation method using five iterations and six subsets. Images were normalised and corrected for decay, dead-time and random events producing an image with 283 mm isotropic voxels. The image was visualised using Vivoquant 1.23 software (InviCRO, Massachusetts, USA).

### Autoradiography

Autoradiography was performed following intravenous injection of 10 MBq ^18^F-FDG 90 min prior to culling. The pancreas was removed and snap-frozen in isopentane at −70°C before cryosectioning (10 µm) at −18°C. Sections were thaw mounted, dried and apposed overnight to a storage phosphor screen before imaging on a Typhoon Trio (GE Healthcare) at 25 µm resolution. Sections were then processed with H&E using standard methods.

### Quantitative PCR

Pancreatic tissue samples were placed in a RNA later solution (QIAGEN, Manchester, UK), stored for at least 24 h at 4°C and then snap-frozen until processed. Total RNA was isolated using the QIAGEN Tissue Lyser and QIAGEN RNeasy kits. cDNA was synthesised from 1 μg of RNA using a QPCR cDNA Synthesis Kit (Applied Biosystems, Paisley, UK) and analysed by quantitative real-time PCR on a 7900 HT real-time PCR system using relative quantification (ΔΔCt) with the Taqman gene expression assays (Applied Biosystems). FAM-labelled assays are listed in online supplementary methods.

### Histology and immunohistochemistry

**Figure 1:**
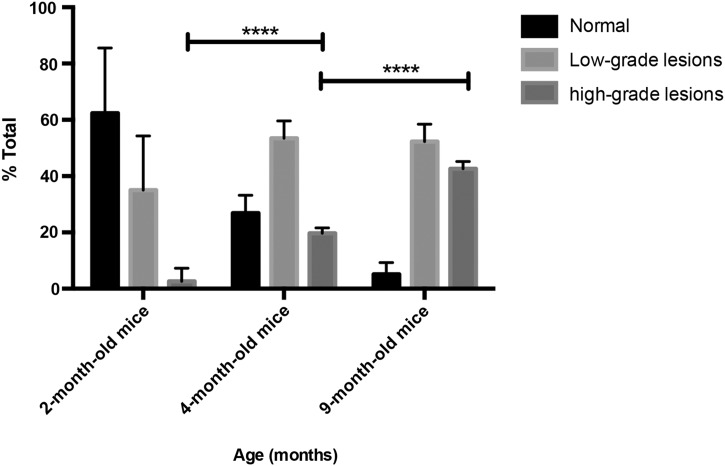
Histological progression of mPanIN in p48Cre;LSL-Kras^G12D^ mice. Percentage of normal (including % of reactive ducts) and neoplastic ducts in low-grade (sum of mPanIN 1 and 1A) and high-grade (sum of mPanIN 2 and 3) lesions in mice with an average age of 2 (n=6), 4 (n=4) and 9 months (n=6).^21^ Mean±SEM; *p<0.05, **p<0.01, ***p<0.001. n, number of animals; PanIN, pancreatic intraepithelial neoplasia.

Sections of formalin-fixed paraffin-embedded tissue were stained with H&E, anti-carbonic anhydrase IX (CAIX) rabbit polyclonal antibody (1:250 dilution) (Santa Cruz, sc-25600, Texas, USA) and with anti-CD31 (1:50 dilution) rat monoclonal antibody (BD Biosciences, Ref. 553370, Oxford, UK). For quantification of mPanIN, pancreata were sectioned at 100 μm intervals and individual mPanIN lesions were counted (figure 1).^21^ mPanIN lesions were classified as low grade (sum of mPanIN 1 and 1A) and high grade (sum of mPanIN 2 and 3), as described previously.^21^ The Aperio Microvessel Analysis V.1 algorithm was used to analyse CD31 staining (Leica Biosystems, Milton Keynes, UK) and tuned to report longitudinal and transverse cut vessels with a minimum vessel area threshold of 50 μm.

### Statistical analysis and quantification

Results are expressed as mean±SEM, unless stated otherwise. Statistical significance was tested using Prism V.6 (GraphPad Software, San Diego, USA) with one-way analysis of variance (ANOVA) (Tukey's post hoc test) or Kruskal–Wallis test when ANOVA assumptions were not met (Dunn's post hoc test).

## Results

### Glucose uptake in the mouse model and in humans

We used KPC mice that carry K-ras and p53 mutations, leading to early onset of PDA, and KC mice, which have only the K-ras mutation and which show slower progressing lesions and develop PDA later in life.^21^ ^22^ As in high-risk individuals, the disease burden in the KC mice increased with time (figure 1), with mice at 2 months having mainly normal tissue (∼60%) and low-grade mPanIN (∼40%), at 4 months mainly low-grade mPanIN and at 9 months equal amounts of low-grade and high-grade mPanIN. Acute pancreatitis was induced in wt mice by intraperitoneal injections of caerulein.^25^ Images and tissue extracts for metabolite and enzymatic analysis were acquired from the whole pancreas, and therefore, reflected the increasing disease burden as the animals aged.

**Figure 2:**
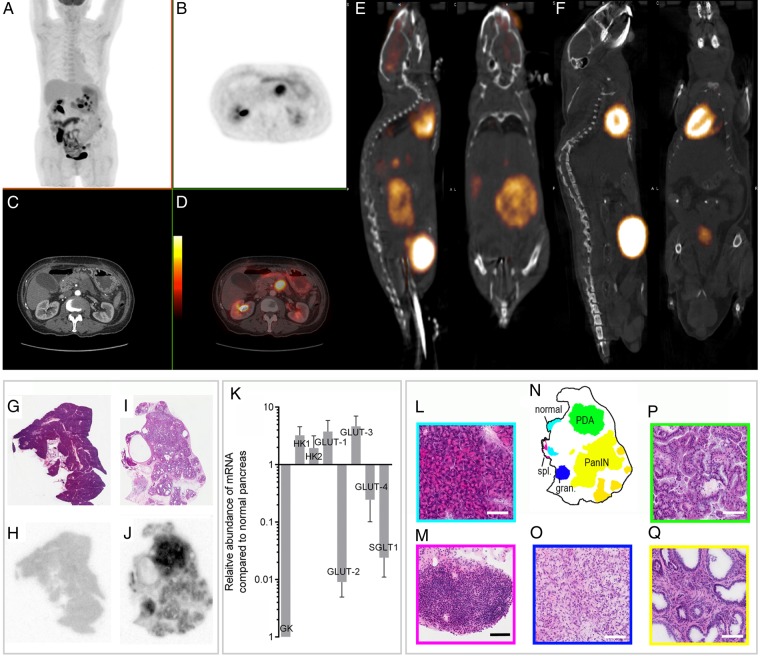
PET-CT images of pancreatic cancer in a human and in a LSL-Kras^G12/D+;^LSL-Tpr53^R172H/+;^Pdx-1-Cre (KPC) mouse and autoradiographic analysis of ^18^F-fluorodeoxyglucose (FDG) uptake in a 9-month-old control (wild-type (wt) mouse, and in a 13-month-old LSL-Kras^G12D/+^- p48^Cre/+^ (KC) mouse with mPanIN lesions and a pancreatic ductal adenocarcinoma (PDA) tumour and changes in hexokinase and glucose transporter expression in PDA-containing pancreas compared with normal pancreas. Clinical images of a 70-year-old woman with metastatic adenocarcinoma of the body of the pancreas and biopsy proven peritoneal and liver metastases. Coronal maximum intensity projection image (A) and axial (B) ^18^F-FDG-PET images acquired with time-of-flight imaging at the level of the pancreas showing tracer uptake in the pancreatic tumour with a maximum standardised uptake value (SUVmax) of 15.4. Axial contrast-enhanced CT (C) and fused PET-CT images (D) with the PET images shown as a false-colour scale superimposed over the grey-scale CT images. Representative ^18^F-FDG PET-CT images from a KPC mouse (E) with histologically confirmed pancreatic ductal adenocarcinoma and a *wt* mouse (F). H&E staining of a representative normal pancreas (G) with corresponding ^18^F-FDG autoradiography (H). H&E staining of a diseased pancreas from a KC mouse (I) and corresponding ^18^F-FDG autoradiography (J). The signal intensities in (H) and (J) are comparable. Areas of distinct histopathology corresponding to the tissue sections in (I) and (J) are shown in (N). At high (20×) magnification, normal pancreatic tissue (L), splenic lymphoid tissue (spl.) (M), granuloma tissue (gran.) (O), pancreatic ductal adenocarcinoma (P) and mouse pancreatic intraepithelial neoplasia^14^ are shown. Horizontal bars represent 100 µm. Overexpression of GLUT-1, GLUT-3, hexokinase 1, hexokinase 2 and underexpression of glucokinase, GLUT-2, GLUT-4 and SGLT1 in PDA-containing pancreas (n=5) compared with normal pancreas (n=4), measured by quantitative real-time-PCR (K). n, number of animals. PanIN, pancreatic intraepithelial neoplasia.

As in the human disease, PDA in these mice showed high levels of ^18^FDG uptake in PET images, reflecting increased glucose uptake and phosphorylation (figure 2). Autoradiography of excised pancreas sections showed that increased FDG uptake was confined to regions containing mPanIN lesions and PDA (figure 2). A recent study using an inducible Kras^G12D^ model of PDA demonstrated loss of FDG uptake and a decrease in glucose uptake and lactate secretion following loss of Kras^G12D^ expression.^28^ This study also showed a decrease in expression of the glucose transporter GLUT1 and in the hexokinases HK1 and HK2. Consistent with this previous study, we observed increased expression of the glucose transporters GLUT1 and GLUT3 and the hexokinases HK1 and HK2 in PDA compared with normal tissue (figure 2).

### Changes in alanine and lactate concentrations and LDH and ALT activities with disease progression

**Figure 3:**
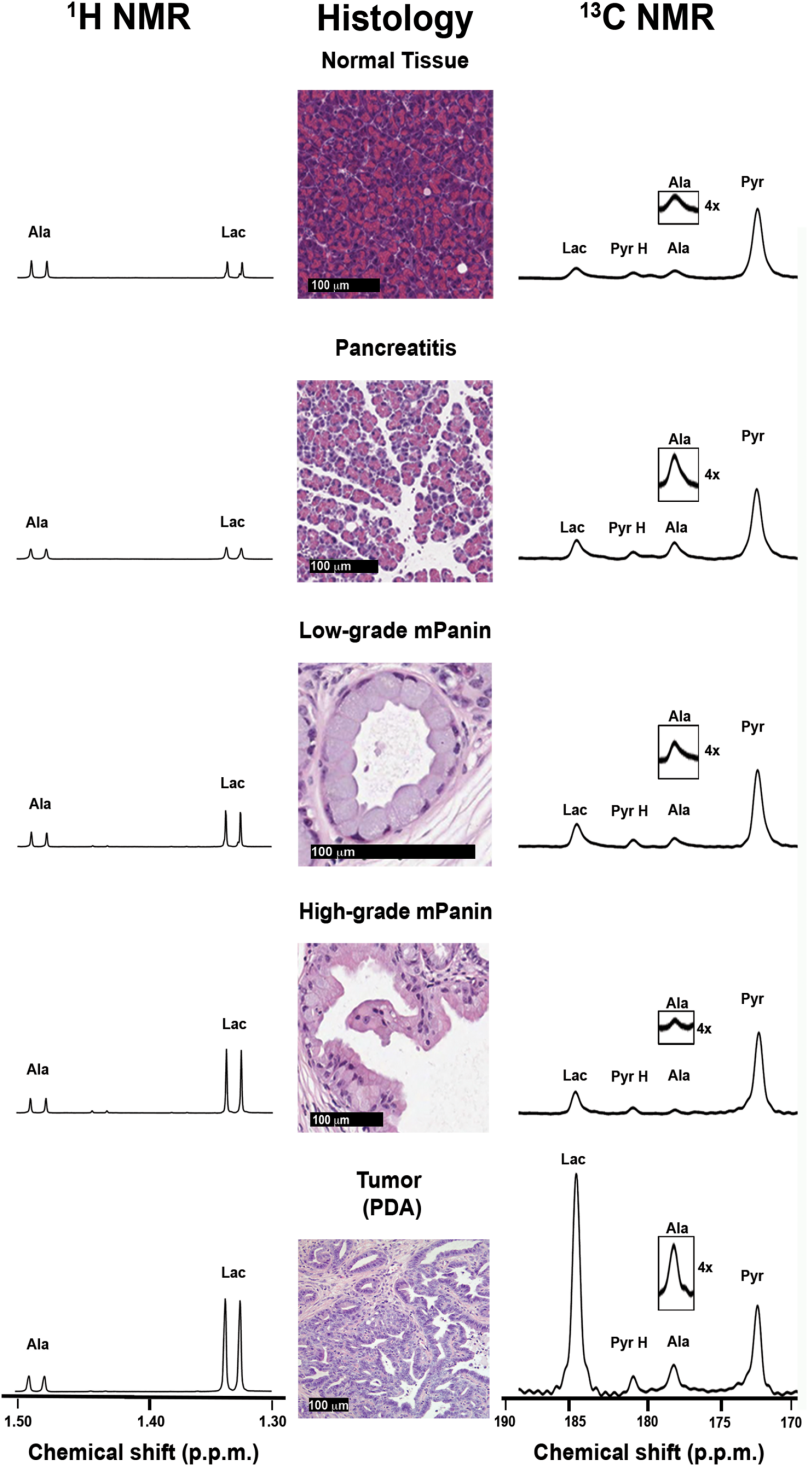
^13^C and ^1^H MR spectra acquired at different stages of disease progression. The predominant lesion present is indicated in the tissue sections obtained post mortem. The ^1^H spectra were acquired from pancreatic tissue extracts and the ^13^C spectra were acquired in vivo following injection of hyperpolarised [1-^13^C]pyruvate. In each condition, alanine and lactate resonances are plotted on the same scale, normalised to the alanine integral in the ^1^H spectra and to the pyruvate peak in the ^13^C spectra. Ala, alanine; Lac, lactate; Pyr, pyruvate; Pyr H, pyruvate hydrate.

**Figure 4:**
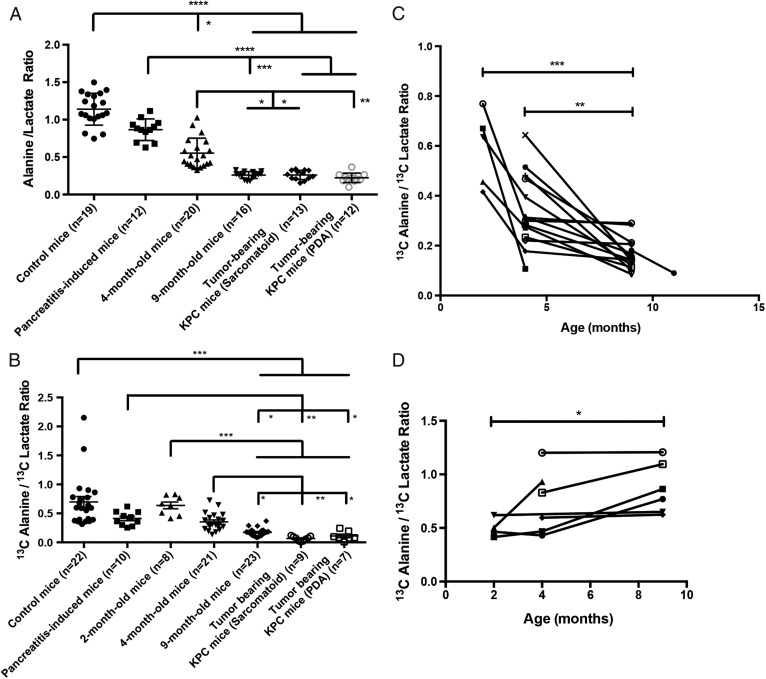
Alanine/lactate concentration ratios measured by ^1^H NMR in pancreatic tissue extracts (A) and the corresponding hyperpolarised [1-^13^C]alanine/[1-^13^C]lactate signal ratios observed in ^13^C chemical shift images of the pancreas (B) of control mice between 2 and 9 months; KC mice at 2, 4 and 9 months and tumour-bearing KPC mice. Changes in the [1-^13^C]alanine/[1-^13^C]lactate signal ratios in individual KC (n=16) (C) and control mice (n=7) (D) at the indicated ages. Mean±SEM. *p<0.05, **p<0.01, ***p<0.001, ****p<0.0001. n, number of animals. PDA, pancreatic ductal adenocarcinoma.

The alanine/lactate concentration ratio, measured in tissue extracts, showed a significant decrease with disease progression (figures 3 and 4A), due primarily to an increase in lactate concentration, which was consistent with the increased FDG uptake in mPanIN and PDA lesions (figure 2) and indicates an increase in glycolytic flux (table 1). There was also a progressive increase in LDH activity (EC 1.1.1.27) (table 1). Decreases in glucose uptake, lactate production and LDHA expression have been reported previously following loss of Kras^G12/D^ expression in a similar mouse model of the disease.^28^ There were no significant changes in the mean vascular density with disease progression (see online supplementary figure S1A), indicating that these metabolic changes are unlikely to be explained by changes in tissue perfusion. We also observed very similar metabolic profiles in sarcomatoid and PDA tumours (see figure 4), which have different morphology, with sarcomatoid tumours being well-vascularised and stromal deficient. 29 There was, however, higher expression of CAIX in high-grade lesions (see online supplementary figure S1B-D).^30^ There were no significant differences in the concentration ratio in the various mouse strains that do not develop disease (see online supplementary figure S2 and table 2) nor in the activity of LDH (table 2). There were also no significant differences in the alanine/lactate concentration ratios between the PDA tumours that developed in KC mice and those that developed in KPC mice (see online supplementary figure S2), demonstrating that the metabolic differences could not be attributed to strain differences.

**Table 2:** Lactate and alanine concentrations and lactate dehydrogenase activities measured in the pancreas of the different mouse strains used in this study that do not develop disease

Tissues	Alanine (μmol/g of wet tissue)	Lactate (μmol/g of wet tissue)	Lactate dehydrogenase activity (mU/mg of protein) (n=3)
Wt (C57BL/6) (n=5)	3.81±0.46† (*)	3.00±0.31† (*)	204.35±53.10
p48^Cre/+^ (n=4)	1.30±0.42	1.07±0.34	369.6±80.2
LSL-Kras^G12D/+^ (n=4)	1.16±0.28	1.12±0.26	229.88±20.50
Pdx-1-Cre (PC) (n=6)	0.93±0.06	0.95±0.13	222.54±55.93

Mean±SEM.

*p<0.05.

†Significantly higher compared with PC.

n, number of animals; wt, wild-type.

### Imaging disease progression with hyperpolarised [1-^13^C]pyruvate

**Figure 5:**
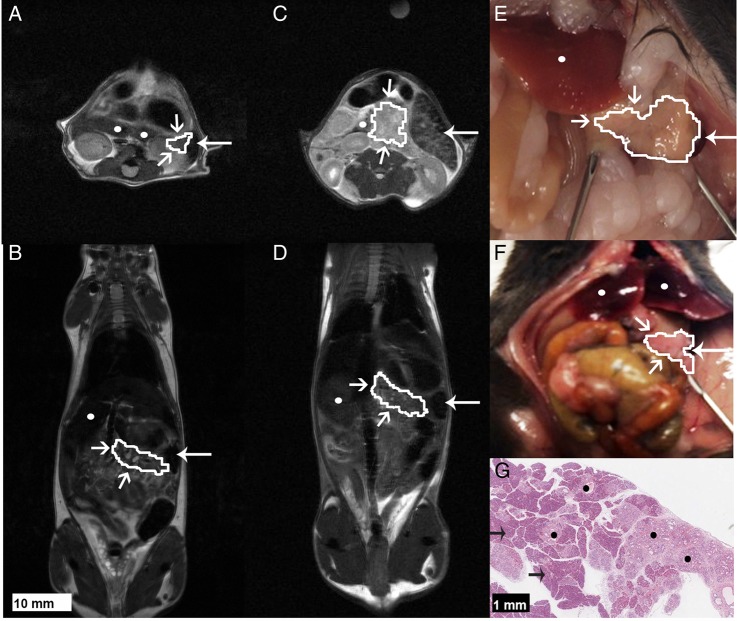
Identification and anatomical location of the pancreas in a wild-type (*wt*) mouse (A), a 9-month-old LSL-Kras^G12D/+^- p48^Cre/+^ (KC) mouse (B), a LSL-Kras^G12/D+;^LSL-Tpr53^R172H/+;^Pdx-1-Cre (KPC) mouse with a pancreatic ductal adenocarcinoma tumour (C) and a 4-month-old KC mouse (D), in axial (A and C) and coronal (B and D) T_2_-weighted ^1^H images and at necropsy. Necropsy of the *wt* mouse (E) from which the image shown in (A) was acquired and the corresponding necropsy (F) and histology (G) of the 4-month-old KC mouse from which the image shown in (D) was acquired. Consistent attachment of the pancreas to the spleen was identified by MRI and at necropsy. The pancreas, or tumour in the case of (C), are demarcated by a white line. Open arrows indicate normal and diseased pancreas and tumour; white filled arrows indicate spleen; white circles indicate liver; black arrows indicate areas of normal pancreas and black circles indicate examples of areas of low-grade mPanIN.

**Figure 6:**
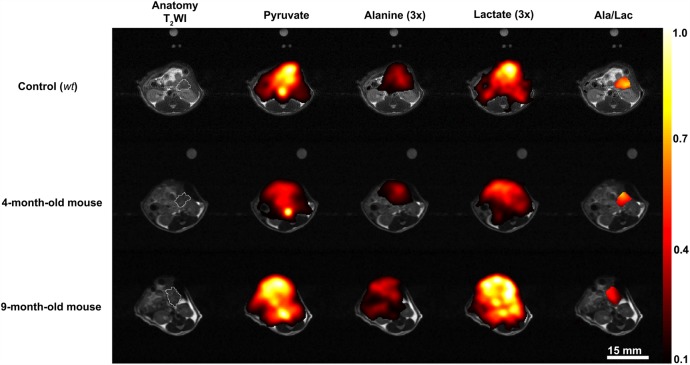
Representative ^13^C spectroscopic images showing the spatial distribution of labelled pyruvate, lactate and alanine in control (wild-type (*wt*)), 4-month-old and 9-month-old LSL-Kras^G12D/+^- p48^Cre/+^ (KC) mice. The lactate, alanine and the alanine/lactate ratio are displayed as voxel intensities relative to the maximal pyruvate signal. In the case of alanine and lactate, these have been increased by a factor of 3 to aid visualisation. The [1-^13^C]alanine/[1-^13^C]lactate signal ratio is shown for a region of interest that encompasses the pancreas. The location of the pancreas was identified in the T_2_-weighted ^1^H images, which are shown in grey scale. The pancreas is outlined in white on the anatomical image. A fiducial marker was included to serve as a reference. The colour scales represent arbitrary linearly distributed intensities for the hyperpolarised images.

^13^C CSI were acquired 20±2 s after intravenous injection of hyperpolarised [1-^13^C]pyruvate from an axial 4–8-mm-thick slice through the pancreas. Slice location was determined from high-resolution T_2_-weighted ^1^H images (figure 5). We observed signals from [1-^13^C]pyruvate, and from [1-^13^C]lactate and [1-^13^C]alanine, which are formed by exchange of the hyperpolarised ^13^C label between the injected pyruvate and the endogenous lactate and alanine pools, respectively (figure 6).^31^ ^32^ The [1-^13^C]alanine/[1-^13^C]lactate signal ratio, which had a coefficient of variation of 20.9% (see online supplementary table S1), showed the same decrease with disease progression as the alanine/lactate concentration ratio (figure 4B). With both measurements, tumour-bearing mice and 9-month-old KC mice, which had a significant amount of high-grade mPanIN (figure 1), could be distinguished from 4-month-old KC mice and from control wt mice and wt mice with induced pancreatitis. The capability of the technique to distinguish between low-grade mPanIN and high-grade mPanIN and tumour became even clearer when 2-month-old KC mice were imaged as models of low-grade PanIN (figure 4B) as these animals have very low levels of high-grade mPanIN (figure 1).^21^ Similar but less marked trends were observed with disease progression in the [1-^13^C]alanine/[1-^13^C]pyruvate and [1-^13^C]lactate/[1-^13^C]pyruvate signal ratios (see online supplementary figure S3). The corresponding inverse trends were observed in the lactate/alanine concentration and [1-^13^C]lactate/[1-^13^C]alanine signal ratios (see online supplementary figure S3). There were no significant differences in the hyperpolarised [1-^13^C]alanine/[1-^13^C]lactate signal ratios in those strains that do not develop disease (see online supplementary figure S2).

In order to determine whether disease progression could be followed in the same individual, we imaged KC mice (n=16), and a cohort of control littermates (n=7), at 2, 4 and 9 months of age. Disease progression was confirmed in another group of KC mice (n=16) where pancreas histology was assessed by an independent blinded observer (figure 1). The higher percentage of high-grade lesions found in the 4-month-old and 9-month-old KC mice, compared with that observed previously, may be explained by the different promoter (p48) used here to drive Cre recombinase expression.^21^ Imaging of these animals, following injection of hyperpolarised [1-^13^C]pyruvate, showed that there was a 44% and a 71% decrease in the [1-^13^C]alanine/[1-^13^C]lactate signal ratio between 2-month-old and 4-month-old and 9-month-old mice, respectively, as well as a 50% decrease between 4-month-old and 9-month-old KC mice (figure 4C), which was consistent with the measurements on groups of animals (figure 4B). Control littermates (LSL-Kras^G12D/+^; p48^Cre^; Pdx-1-Cre (PC) and C57BL/6 wt) showed either no change in the ratio or an increase (figure 4D).

### Measurements of ^13^C labelling in pancreas extracts

Imaging of lactate and alanine labelling in the pancreas in vivo was validated by measuring ^13^C labelling of alanine and lactate in rapidly excised and freeze-clamped pancreas and by measuring label exchange in cell-free pancreas homogenates. Partitioning of ^13^C label between injected, non-hyperpolarised, [3-^13^C]pyruvate, and endogenous alanine and lactate was reproduced in cell-free pancreatic tissue extracts, where the decrease in the [3-^13^C]-labelled alanine/lactate signal ratio with disease progression was similar to that measured in freeze-clamped tissue extracts prepared 20 s after intravenous injection of [3-^13^C]pyruvate (table 3) and similar also to the hyperpolarised [1-^13^C]alanine/[1-^13^C]lactate ratio measured in vivo in 9-month-old KC and tumour-bearing mice (figure 4B). The lower ratio observed in vivo for normal tissue, pancreatitis and, to a lesser extent, for 4-month-old mice may reflect partial volume effects, where tissue outside the pancreas was imaged.

**Table 3:** Partition of ^13^C label between alanine and lactate following incubation of [3-^13^C]pyruvate with pancreatic tissue homogenates and in pancreatic tissues in vivo following intravenous injection of [3-^13^C]pyruvate

Pancreatic tissue type	Concentrations of added metabolites		[3-^13^C]alanine/[3-^13^C]lactate ratio in pancreatic homogenates	^13^C-alanine/^13^C-lactate ratio in vivo (20 s after intravenous injection of [3-^13^C]pyruvate)
	Alanine (mM)	Lactate (mM)		
Control	2	2	3.34	2.28
Induced pancreatitis	1	1	1.33	0.56
4-month-old mice	1	2	0.85	0.62
9-month-old mice	1	4	0.11	0.17
Tumour (PDA)	2	9	0.04	0.09

Partitioning of the ^13^C label between alanine and lactate was measured in pancreatic tissue homogenates using dynamic ^1^H NMR measurements and by ^13^C NMR in pancreatic tissue that was rapidly excised 20 s after intravenous injection of 0.3 mL of 82 mM [3-^13^C]pyruvate.

PDA, pancreatic ductal adenocarcinoma.

## Discussion

We have shown that imaging exchange of hyperpolarised ^13^C label between injected [1-^13^C]pyruvate and the endogenous alanine and lactate pools can be used to detect and follow the progression of pancreatic precursor lesions, differentiating normal pancreas, pancreatitis and tissue with predominantly low-grade mPanIN from tissue with predominantly high-grade mPanIN and tumour. This distinction is the most important clinically since patients with high-grade lesions or early-stage PCa could be offered potentially curative surgery.^11^ Differentiation was most clear for KC mice at 2 months of age, where there was largely normal tissue and low-grade mPanIN present (figure 1), in which was no overlap of the hyperpolarised [1-^13^C]alanine/[1-^13^C]lactate signal ratios with the ratios observed in 9-month-old and tumour-bearing animals (figure 4B). However, although we could distinguish in these inbred mouse strains pancreas with predominantly low-grade mPanIN from tissue with predominantly high-grade mPanIN and tumour, it is unlikely that these thresholds for the ^13^C-labelled alanine/lactate ratio would be preserved or indeed consistent in an outbred human population. Instead, we envisage that the technique would be used in human individuals at high risk of developing PCa to look for evidence of the presence of disease and subsequently to monitor disease progression. The feasibility of such an approach was demonstrated by following disease progression in individual animals (figure 4C).

The decrease in the [1-^13^C]alanine/[1-^13^C]lactate signal ratio can be explained by increases in lactate concentration (figure 3) and LDH activity and a decrease in ALT activity (EC 2.6.1.2) (table 1).^19^ ^31^ Alanine concentration also affects ^13^C hyperpolarised label exchange between [1-^13^C]pyruvate and alanine (see online supplementary table S2); however, the alanine concentrations were largely unchanged (table 1). Increased LDH activity has been reported in human PCa and decreases in glucose uptake and lactate production and in GLUT1, HK1, HK2 and LDHA expression have been reported previously following loss of Kras^G12/D^ expression in a similar mouse model of PCa.^28^ ^33^ Although we observed an increase in CAIX expression, which is regulated by HIF1α, HIF1α is not thought to play a significant role in glycolytic enzyme expression in this model of PCa.^28^ ^30^ Instead, the MAPK pathway and Myc-directed transcription are thought to play key roles.^28^ Similar patterns of lactate and alanine labelling, following injection of hyperpolarised [1-^13^C]pyruvate, have been observed previously in prostate tumours, where increased lactate labelling was observed with increasing histological grade and in a c-Myc-driven liver cancer model, where higher alanine labelling was observed in pre-cancerous lesions and higher lactate labelling in the resulting tumours.^20^ ^34^

Several promising molecular imaging probes for detection of PanIN lesions have been described, including Claudin-4 and cathepsin-activatable near-infrared probes and a single-photon emission CT (SPECT) probe, based on Plectin-1, which detected primary pancreatic tumours and metastatic foci in orthotopic mouse models of PDA.^35–37^ The ^13^C-MRSI experiment described here, however, has several potential advantages. In contrast to imaging with NIR probes, which may be limited by a requirement to use invasive and operator-dependent procedures, such as endoscopy or laparotomy, the technique could be used to produce non-invasive three-dimensional metabolic maps of the pancreas, at depth, which should allow more ready detection of diseased areas. Unlike imaging with PET or SPECT probes, the technique does not use ionising radiation and therefore could be used to screen and follow-up high-risk patients over a prolonged period of time.^37^ Recent observations suggest that at least 10 years are needed for the initiating mutation to evolve into invasive carcinoma within the high-grade PanIN lesion and an additional 5–6 years are required before cancer cells acquire metastatic capability.^38^ The technique may also offer opportunities for ultrasound (US)-guided targeted endoscopic biopsies using fused US-MRSI images.

A limitation of the current study was the extent of disease in the mouse model, where in 9-month-old KC mice high-grade mPanIN occupied ∼40% of the pancreas. However, the human pancreas is considerably larger, measuring approximately 12–20 cm×2 cm×2 cm as compared to ∼1.8 cm×∼5 mm (craniocaudal axis)×∼3 mm (anteroposterior axis) in the mouse, and in individuals with FPC PanIN lesions consist of small, generally <5 mm, intraductal lesions, which in high-risk individuals are focally distributed at a rate of 1.51 lesions/cm^2^.^39–43^ Since the voxel size used in the first clinical study in prostate cancer was 7×7×7 mm^3^, which it may be possible to improve on with higher levels of polarisation, then we believe that both the image resolution and the disease burden should be sufficient to detect disease progression in the clinic.^44^ ^45^ The main factor limiting translation to the clinic is the short half-life of the hyperpolarised ^13^C label. Nevertheless, initial results from the first clinical trial have shown this to be sufficient to probe lesions in the prostate.^44^

The absolute signal intensities from pyruvate, alanine and lactate are dependent on a number of factors, including the degree of polarisation of the injected pyruvate, transit time, amount of pyruvate delivered to the tissue and the variable rate of loss of polarisation in all three species. However, by measuring the ratio of the signal in alanine and lactate, which depends mainly on the relative activities of LDH and ALT and the alanine and lactate pool sizes, these factors are largely corrected for.

In summary, improved diagnostic tools for screening and follow-up of individuals at high risk of developing PCa represent a clear and unmet clinical need.^11–13^ We have shown here that imaging exchange of hyperpolarised ^13^C label between injected [1-^13^C]pyruvate and the endogenous alanine and lactate pools, in well-established and realistic mouse models of the disease, can be used to non-invasively detect and follow progression of pancreatic preneoplastic lesions. The technique may provide an improved diagnostic and screening tool for individuals at high risk of developing PCa, enabling not only a better risk stratification but also earlier curative intervention with potential improvements in overall prognosis and patient survival. However, the true potential of the technique can only be established in clinical studies on patients with PCa and on individuals at risk of developing the disease.

## Supplementary Material

Web supplement
