# Supplementary material for: MRI with hyperpolarised [1-13C]pyruvate detects advanced pancreatic preneoplasia prior to invasive disease in a mouse model
Source: Gut. 2015 Sep 7;65(3):465–75. doi: 10.1136/gutjnl-2015-310114 (PMC4789827; doi:10.1136/gutjnl-2015-310114)
Supplement: Web supplement [file gutjnl-2015-310114-s1.pdf]

SUPPLEMENTARY INFORMATION

Magnetic resonance imaging with hyperpolarized [1-^{13}C]pyruvate detects advanced pancreatic preneoplasia prior to invasive disease in mouse model

Eva M. Serrao[1, 2], Mikko I. Kettunen[1,2,3], Tiago B. Rodrigues[1,2], Piotr Dzien[1,2], Alan J. Wright[1, 2], Aarthi Gopinathan[1], Ferdia A. Gallagher[1,4], David Y. Lewis[1, 2], Kristopher K. Frese[5], Jaime Almeida[1], William J. Howat[1], David A. Tuveson[6], Kevin M. Brindle[1,2]†

[1]Cancer Research UK Cambridge Institute, University of Cambridge, UK

[2]Department of Biochemistry, University of Cambridge, UK

[3]A.I.Virtanen Institute for Molecular Sciences, University of Eastern Finland, Kuopio, Finland

[4]Department of Radiology, University of Cambridge, Cambridge, UK

[5]Princess Margaret Cancer Centre, 610 University Ave, Toronto, ON, M6C 1K6, Canada

[6]Cold Spring Harbor Laboratory, NY, USA

SUPPLEMENTARY METHODS

Quantitative PCR

FAM labeled assays were used in conjunction with TaqmanFast mastermix 384-wellplates. Actin was used as the endogenous control.

List of Taqman probes:

Gene	*Gene Name*	*Abbreviation*	*Taqman Code*
Actb	actin		Mm00607939_s1
Gck	glucokinase	GK	Mm00439129_m1
Hk1	hexokinase 1	HK1	Mm00439344_m1
Hk2	hexokinase 2	HK2	Mm00443385_m1
Slc2a1	solute carrier family 2 (facilitated glucose transporter), member 1	GLUT-1	Mm00441473_m1
Slc2a2	solute carrier family 2 (facilitated glucose transporter), member 2	GLUT-2	Mm00446229_m1
Slc2a3	solute carrier family 2 (facilitated glucose transporter), member 3	GLUT-3	Mm00441483_m1
Slc2a4	solute carrier family 2 (facilitated glucose transporter), member 4	GLUT-4	Mm00436615_m1
Slc5a1	solute carrier family 5 (sodium/glucose cotransporter), member 1	SGLT1	Mm00451203_m1

SUPPLEMENTARY TABLES AND FIGURES

Supplementary Table S1. Mean coefficient of variation of the hyperpolarized [1-^{13}C]Alanine/[1-^{13}C]Lactate signal ratios in control, KC and KPC mice following repeat measurements.

Mouse no	Measurement 1	Measurement 2	Mean	SD	COV
1	0.289	0.281	0.285	0.006	0.021
2	0.165	0.091	0.128	0.052	0.408
3	0.173	0.154	0.163	0.013	0.082
4	0.149	0.072	0.111	0.055	0.496
5	0.729	0.568	0.648	0.113	0.175
6	0.176	0.243	0.210	0.048	0.227
7	0.174	0.232	0.203	0.041	0.204
8	0.212	0.231	0.222	0.013	0.059
				Mean COV:	0.209

Hyperpolarized [1-^{13}C]alanine/[1-^{13}C]lactate signal ratios observed in ^{13}C chemical shift images of the pancreas from control (n=1), 9 month-old KC mice (n=3) and KPC mice with PDA tumors (n=4), acquired 24 hours apart. n= number of animals.

Supplementary Table S2. Rate of [3-^{13}C]alanine formation and alanine transaminase activity.

Rate of [3-^{13}C]alanine formation and alanine transaminase activity		
Tissue type	Concentration of alanine (mM)	Rate (x10^{-7}) M/s
9 month-old KC mice pancreas	1	2.76
9 month-old KC mice pancreas	5	3.43
9 month-old KC mice pancreas	10	5.15
Tissue type	ALT activity at time point 1 (mU/mL)	ALT activity at time point 2 (mU/mL)
9 month-old KC mice pancreas	126.67	98.25

The rates ^{13}C label exchange between 10 mM [3-^{13}C]pyruvate and the indicated unlabeled alanine concentrations were measured dynamically by ^{1}H NMR. Enzyme activity was determined by spectrophotometric assay in the same extract at the start (time point 1) and at the end (time point 2) of the NMR spectral acquisition. There was some loss of enzyme activity during the incubation.

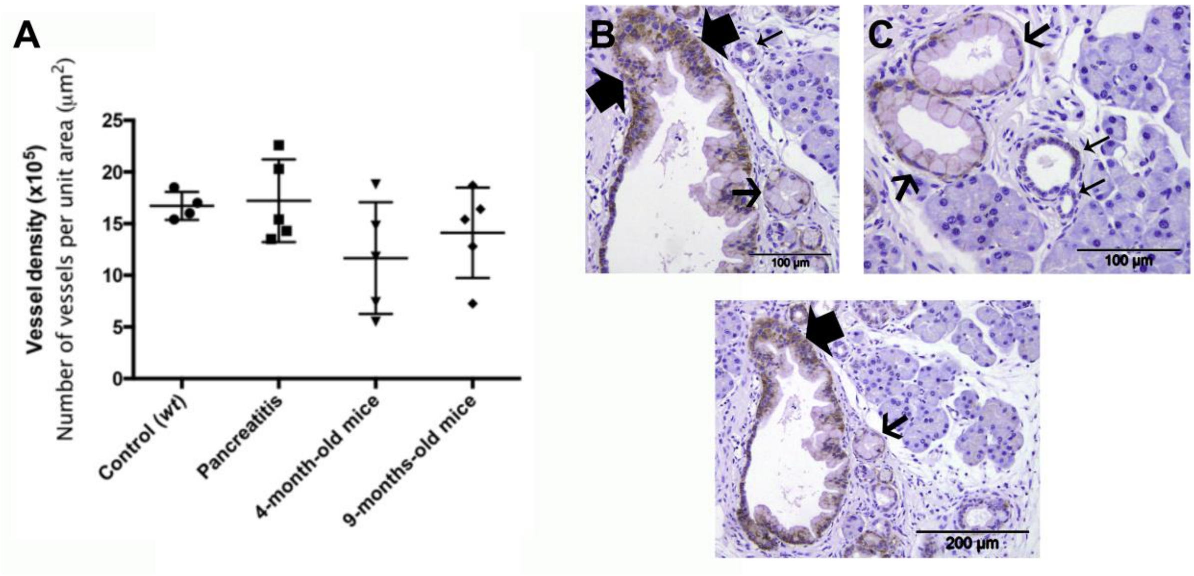

Supplementary Figure S1. Quantitation of blood vessel density in pancreatic tissues from *wt* controls (n=4), *wt* mice with caerulein-induced pancreatitis (n=5), PanIN tissues from 4 (n=5) and 9 (n=5) month-old KC mice (A). Immunohistochemical staining for CAIX expression counterstained with H&E in representative sections from 9 month-old KC mice (B-D). Thick arrows indicate high-grade mPanIN; thin arrows indicate low-grade lesions and small arrows indicate normal ducts.

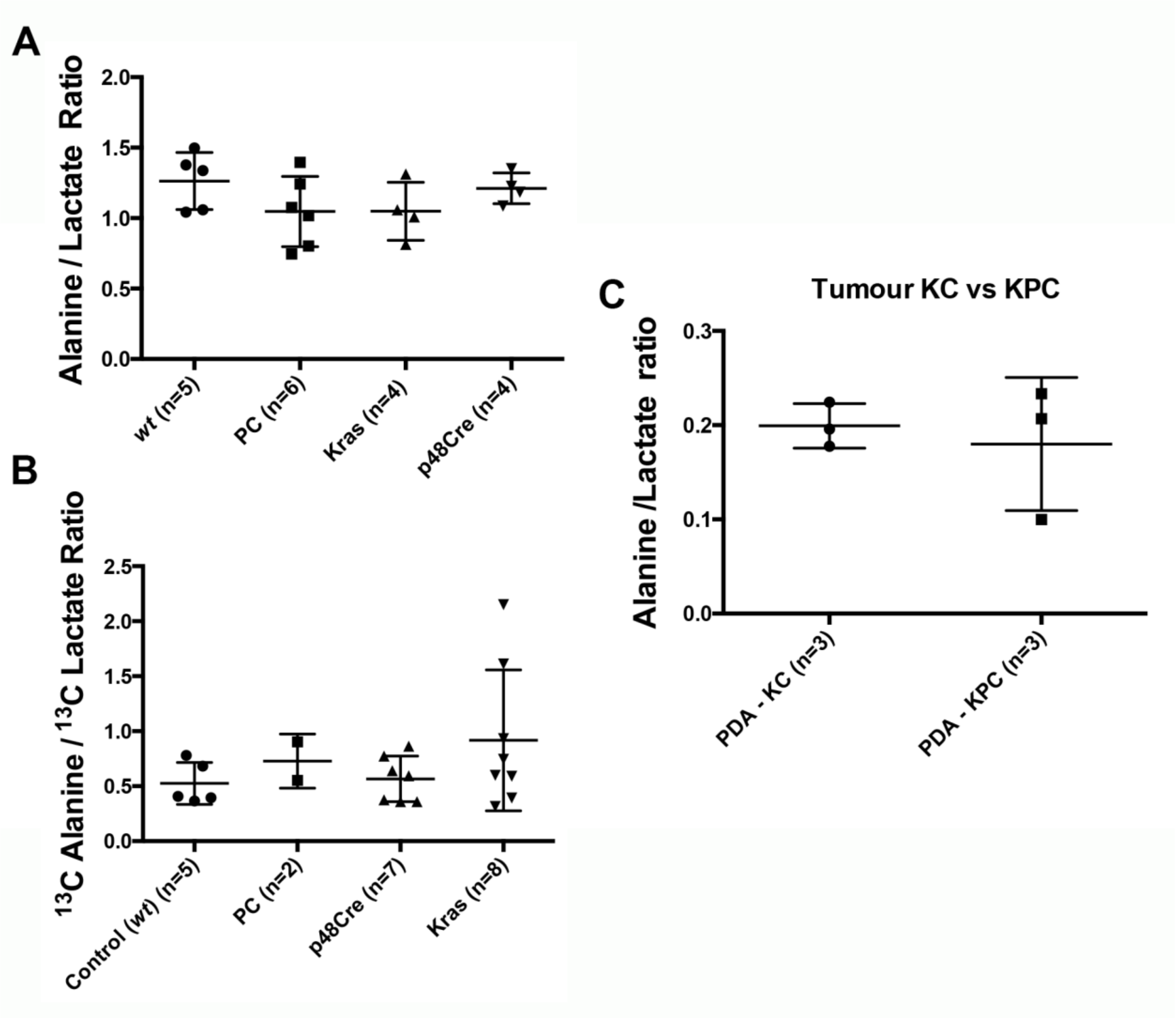

Supplementary Figure S2. Alanine/lactate concentration ratios measured by ^{1}H NMR in pancreatic tissue extracts prepared from mice with the indicated genotype, in which disease does not develop (A), and the corresponding hyperpolarized [1-^{13}C]alanine/[1-^{13}C]lactate signal ratios observed in ^{13}C chemical shift images of the pancreas of these animals (B). Alanine/lactate concentration ratios measured by ^{1}H NMR in PDA tumors from KC and KPC mice (C). Mean ± S.E.M.; * $p < 0.05$, ** $p < 0.01$, *** $p < 0.001$. n= number of animals.

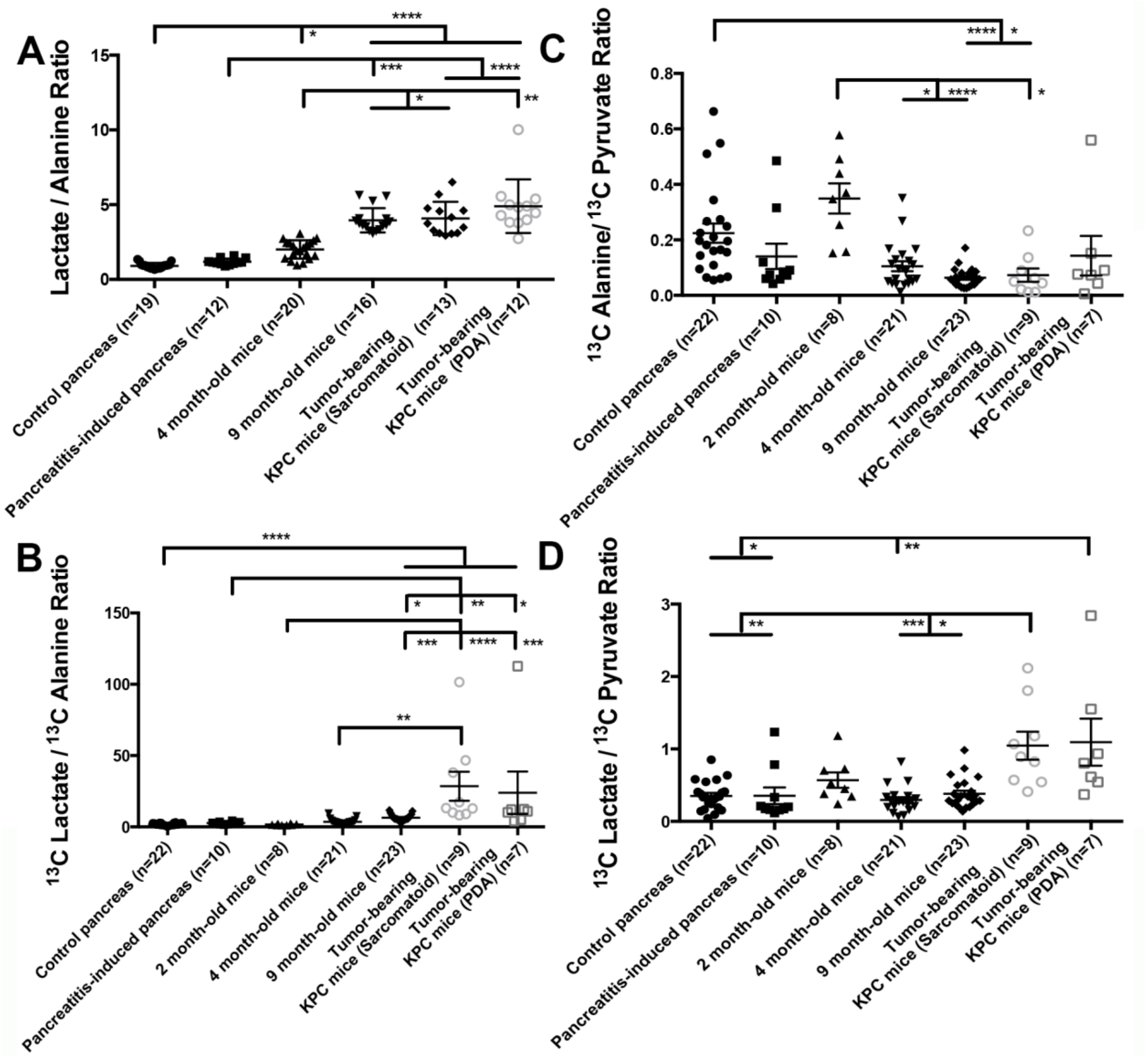

Supplementary Figure S3. Lactate/alanine concentration ratios measured by ^{1}H NMR in pancreatic tissue extracts (A) and the corresponding hyperpolarized [1-^{13}C]lactate/[1-^{13}C]alanine signal ratios observed in ^{13}C chemical shift images of the pancreas (B). Hyperpolarized [1-^{13}C]alanine/[1-^{13}C]pyruvate (C) and [1-^{13}C]lactate/[1-^{13}C]pyruvate (D) signal ratios observed in ^{13}C chemical shift images of the pancreas. Mean ± S.E.M.; * p <0.05, ** p <0.01, *** p <0.001, **** p <0.0001.
